# The *Drosophila* nicotinic acetylcholine receptor subunits Dα5 and Dα7 form functional homomeric and heteromeric ion channels

**DOI:** 10.1186/1471-2202-13-73

**Published:** 2012-06-22

**Authors:** Stuart J Lansdell, Toby Collins, Jim Goodchild, Neil S Millar

**Affiliations:** 1 Department of Neuroscience, Physiology and Pharmacology, University College London, London, UK; 2 Syngenta, Jealotts Hill International Research Centre, Bracknell, Berkshire, UK

## Abstract

**Background:**

Nicotinic acetylcholine receptors (nAChRs) play an important role as excitatory neurotransmitters in vertebrate and invertebrate species. In insects, nAChRs are the site of action of commercially important insecticides and, as a consequence, there is considerable interest in examining their functional properties. However, problems have been encountered in the successful functional expression of insect nAChRs, although a number of strategies have been developed in an attempt to overcome such difficulties. Ten nAChR subunits have been identified in the model insect *Drosophila melanogaster* (Dα1-Dα7 and Dβ1-Dβ3) and a similar number have been identified in other insect species. The focus of the present study is the Dα5, Dα6 and Dα7 subunits, which are distinguished by their sequence similarity to one another and also by their close similarity to the vertebrate α7 nAChR subunit.

**Results:**

A full-length cDNA clone encoding the *Drosophila* nAChR Dα5 subunit has been isolated and the properties of Dα5-, Dα6- and Dα7-containing nAChRs examined in a variety of cell expression systems. We have demonstrated the functional expression, as homomeric nAChRs, of the Dα5 and Dα7 subunits in *Xenopus* oocytes by their co-expression with the molecular chaperone RIC-3. Also, using a similar approach, we have demonstrated the functional expression of a heteromeric ‘triplet’ nAChR (Dα5 + Dα6 + Dα7) with substantially higher apparent affinity for acetylcholine than is seen with other subunit combinations. In addition, specific cell-surface binding of [^125^I]-α-bungarotoxin was detected in both *Drosophila* and mammalian cell lines when Dα5 was co-expressed with Dα6 and RIC-3. In contrast, co-expression of additional subunits (including Dα7) with Dα5 and Dα6 prevented specific binding of [^125^I]-α-bungarotoxin in cell lines, suggesting that co-assembly with other nAChR subunits can block maturation of correctly folded nAChRs in some cellular environments.

**Conclusion:**

Data are presented demonstrating the ability of the *Drosophila* Dα5 and Dα7 subunits to generate functional homomeric and also heteromeric nAChRs.

## Background

Nicotinic acetylcholine receptors (nAChRs) are excitatory neurotransmitter receptors that are found in both vertebrate and invertebrate species. In insects, nAChRs are expressed throughout the nervous system and are the site of action for economically important insecticides such as spinosyns and neonicotinoids [1,2]. Detailed information is available concerning the structure of nAChRs, as a consequence of studies conducted with receptors purified from the electric organ of the marine ray *Torpedo*[3] and from X-ray crystallographic studies conducted with nAChR fragments [4] and also with the closely related acetylcholine binding protein [5]. Nicotinic receptors are assembled from five subunits arranged around a central cation-selective pore [6,7]. Conventional agonists, such as acetylcholine, activate the receptor by binding at an extracellular site located at the interface between two subunits [8], although recent evidence indicates that nAChRs can also be activated by ligands binding to an allosteric transmembrane site [9].

Ten nAChR subunits (Dα1-Dα7 and Dβ1-Dβ3) have been identified in the model insect *Drosophila melanogaster* and a similar number of subunits have been identified in other insect species [1,2]. Despite considerable efforts, there has been only limited success in expressing insect nAChRs in artificial expressions systems [10,11] and, where functional expression has been achieved, ion channel currents have tended to be small or have been generated in response to relatively high agonist concentrations [12-14]. Experimental approaches that have had some success in overcoming problems associated with expression of insect nAChRs include the expression of subunit chimeras containing domains from other neurotransmitter receptors [15], co-expression of insect nAChRs with vertebrate subunits [16,17] or a combination of these approaches [18]. Co-expression with vertebrate nAChR subunits is an approach that has been used in the characterization of nAChR subunits cloned from insect pest species such as the aphid *Myzus persicae*[19,20] and the brown planthopper *Nilaparvata lugens*[21,22]. However, for most insect species for which nAChRs have been cloned, there have been no reports of successful heterologous expression. This includes nAChRs cloned from the honeybee *Apis mellifera*[23-25], diamondback moth *Plutella xylostella*[26,27], house fly *Musca domestica*[28-30], locust *Locusta migratoria*[31], mosquito *Anopheles gambiae*[25], red flour beetle *Tribolium castaneum*[25,32], silkworm *Bombyx mori*[25,33] and tobacco hornworm *Manduca sexta*[34].

RIC-3 is a nAChR-associated molecular chaperone that was originally characterised in the nematode *Caenorhabditis elegans*[35] but has also been identified in several other species, including mammals and insects [36]. It is a transmembrane protein that is able to enhance maturation (folding and assembly) of several nAChR subtypes [36]. For example, co-expression of RIC-3 with the vertebrate nAChR α7 subunit enhances levels of functional expression in *Xenopus* oocytes [35] and is able to facilitate the functional expression of α7 nAChRs in mammalian cell lines that are otherwise non-permissive for expression of α7 [37,38]. In some cell types it has been found that the α7 subunit can be expressed (subunit protein can be detected) but, in the absence of RIC-3, is unable to fold into a conformation that can be detected by radioligand binding or form functional nAChRs [37,38]. In addition, some success has been achieved in overcoming difficulties associated with expression of insect nAChRs by the co-expression with RIC-3 [39,40].

The Dα5, Dα6 and Dα7 subunits of *Drosophila* show close sequence similarity to one another (53-63% amino acid identity [41]) and also have close similarity to the vertebrate nAChR α7 subunit (42-46% amino acid identity [42]). Of the three *Drosophila* subunits, Dα5 and Dα7 have the closest sequence similarity to one another and Dα6 has the highest sequence similarity to the vertebrate α7 [43]. In the present study, we report the molecular cloning of the Dα5 subunit, the only *Drosophila* nAChR subunit for which a full-length cDNA clone was not previously available in our laboratory. Heterologous expression studies with Dα5, Dα6 and Dα7 are described in three host cell types: *Drosophila* S2 cells, human tsA201 cells and *Xenopus* oocytes. Functional expression of several subunit combinations has been achieved in *Xenopus* oocytes and has enabled the pharmacological properties of recombinant nAChRs to be examined. Evidence is provided that demonstrates the ability of subunits to form both homomeric and heteromeric nAChRs. Of particular note is evidence that Dα5 can generate functional homomeric channels and that Dα7 can form both homomeric and heteromeric channels. We are not aware of any previous studies demonstrating the ability of Dα5 and Dα7 subunits to generate such recombinant nAChRs, either with subunits cloned from *Drosophila* or with analogous nAChR subunits from other insect species.

## Results

### Molecular cloning of Dα5

A full-length cDNA encoding the *Drosophila* nAChR Dα5 subunit was isolated from a preparation of *Drosophila* embryo mRNA. The Dα5 cDNA encodes an open reading frame of 807 amino acids corresponding to the previously described Dα5 isoform B [41]. In agreement with previous studies [41], the Dα5 cDNA isolated in this study contains an open reading frame encoding an unusually large N-terminal domain, extending some 300 amino acids upstream of the start methionine in most nAChR subunits.

### Heterologous expression of Dα5 in *Drosophila* and human cell lines

The full-length coding sequence of the Dα5 cDNA was sub-cloned into the *Drosophila* expression vector pRmHa3 (to facilitate expression in *Drosophila* S2 cells) and into pRK5 (to facilitate expression in human tsA201 cells). In cells transfected with pRmHa3-Dα5 or pRK5-Dα5 alone, no evidence of specific high-affinity binding of nicotinic radioligands (^125^I]-α-bungarotoxin, ^3^ H]-epibatidine or ^3^ H]-methyllycaconitine) could be detected. The Dα5 subunit was also co-expressed with an extensive series of *Drosophila* nAChR subunit combinations. Expression studies with more than 100 different subunit combinations (containing between 2 and 10 different *Drosophila* nAChR subunit subtypes) have been examined in our laboratory. However, no specific binding was detected with any these combinations (in the absence of any co-expressed chaperone proteins, see later). To illustrate the extent of these studies, details of all *Drosophila* nAChR subunit combinations containing the Dα5 subunit are listed in Table 1. It is possible that the lack of radioligand binding is a consequence of the expressed subunit proteins failing to undergoing appropriate maturation (folding and assembly) due to a requirement for specific chaperone proteins, as has been reported for other nAChR subunits [37,38], or due to a requirement for additional nAChR subunits.

**Table 1:** **Radioligand binding to *Drosophila* nAChR subunit combinations**

**Subunit combination**	**[**^**125**^**I]-α-BTX Binding**		**[**^**3**^ **H]-epibatidine binding**	
	**- RIC3**	**+ RIC3**	**- RIC3**	**+ RIC3**
Dα5	–	–	–	–
Dα5/Dα1	–	–	–	–
Dα5/Dα2	–	–	–	–
Dα5/Dα3	–	–	–	–
Dα5/Dα4	–	–	–	–
Dα5/Dα6	–	+	–	–
Dα5/Dα7	–	–	–	–
Dα5/Dβ1	–	–	–	–
Dα5/Dβ2	–	–	–	–
Dα5/Dβ3	–	–	–	–
Dα5/Dα1/Dα2	–	–	–	–
Dα5/Dα1/Dβ1	–	–	–	–
Dα5/Dα1/Dβ2	–	–	–	–
Dα5/Dα1/Dβ3	–	–	–	–
Dα5/Dα2/Dβ1	–	–	–	–
Dα5/Dα2/Dβ2	–	–	–	–
Dα5/Dα2/Dβ3	–	–	–	–
Dα5/Dα3/Dβ1	–	–	–	–
Dα5/Dα3/Dβ2	–	–	–	–
Dα5/Dα3/Dβ3	–	–	–	–
Dα5/Dα4/Dβ1	–	–	–	–
Dα5/Dα4/Dβ2	–	–	–	–
Dα5/Dα4/Dβ3	–	–	–	–
Dα5/Dα6/Dα7	–	–	–	–
Dα5/Dβ1/Dβ2	–	–	–	–
Dα5/Dβ1/Dβ3	–	–	–	–
Dα5/Dβ2/Dβ3	–	–	–	–
Dα5/Dα1/Dα2/Dβ1	–	–	–	–
Dα5/Dα1/Dα2/Dβ2	–	–	–	–
Dα5/Dα1/Dα2/Dβ3	–	–	–	–
Dα5/Dα1/Dα3/Dβ1	–	–	–	–
Dα5/Dα1/Dα3/Dβ2	–	–	–	–
Dα5/Dα1/Dα3/Dβ3	–	–	–	–
Dα5/Dα1/Dα4/Dβ1	–	–	–	–
Dα5/Dα1/Dα4/Dβ2	–	–	–	–
Dα5/Dα1/Dα4/Dβ3	–	–	–	–
Dα5/Dα2/Dα3/Dβ1	–	–	–	–
Dα5/Dα2/Dα3/Dβ2	–	–	–	–
Dα5/Dα2/Dα3/Dβ3	–	–	–	–
Dα5/Dα2/Dα4/Dβ1	–	–	–	–
Dα5/Dα2/Dα4/Dβ2	–	–	–	–
Dα5/Dα2/Dα4/Dβ3	–	–	–	–
Dα5/Dα3/Dα4/Dβ1	–	–	–	–
Dα5/Dα3/Dα4/Dβ2	–	–	–	–
Dα5/Dα3/Dα4/Dβ3	–	–	–	–
Dα5/Dα6/Dα7/Dβ1	–	–	–	–
Dα5/Dα6/Dα7/Dβ2	–	–	–	–
Dα5/Dα6/Dα7/Dβ3	–	–	–	–
Dα5/Dβ1/Dβ2/Dβ3	–	–	–	–
Dα5/Dα1/Dα2/Dα3/Dβ1	–	–	–	–
Dα5/Dα1/Dα2/Dα3/Dβ2	–	–	–	–
Dα5/Dα1/Dα2/Dα3/Dβ3	–	–	–	–
Dα5/Dα1/Dα2/Dβ1/Dβ2	–	–	–	–
Dα5/Dα1/Dα3/Dβ1/Dβ2	–	–	–	–
Dα5/Dα1/Dα4/Dβ1/Dβ2	–	–	–	–
Dα5/Dα2/Dα3/Dβ1/Dβ2	–	–	–	–
Dα5/Dα2/Dα4/Dβ1/Dβ2	–	–	–	–
Dα5/Dα3/Dα4/Dβ1/Dβ2	–	–	–	–
Dα5/Dα1/Dα2/Dβ2/Dβ3	–	–	–	–
Dα5/Dα1/Dα3/Dβ2/Dβ3	–	–	–	–
Dα5/Dα1/Dα4/Dβ2/Dβ3	–	–	–	–
Dα5/Dα1/Dβ1/Dβ2/Dβ3	–	–	–	–
Dα5/Dα2/Dα3/Dβ1/Dβ2	–	–	–	–
Dα5/Dα2/Dα4/Dβ1/Dβ2	–	–	–	–
Dα5/Dα2/Dβ1/Dβ2/Dβ3	–	–	–	–
Dα5/Dα3/Dα4/Dβ1/Dβ2	–	–	–	–
Dα5/Dα3/Dβ1/Dβ2/Dβ3	–	–	–	–
Dα5/Dα1/Dα2/Dα3/Dα4/Dβ1	–	–	–	–
Dα5/Dα1/Dα2/Dα3/Dα4/Dβ2	–	–	–	–
Dα5/Dα1/Dα2/Dα3/Dα4/Dβ3	–	–	–	–
Dα5/Dα6/Dα7/Dβ1/Dβ2/Dβ3	–	–	–	–
Dα5/Dα1/Dα2/Dα3/Dα4/Dα6/Dα7/Dβ2	–	–	–	–
Dα5/Dα1/Dα2/Dα3/Dα4/Dα6/Dα7/Dβ1/Dβ2	–	–	–	–
Dα5/Dα1/Dα2/Dα3/Dα4/Dα6/Dα7/Dβ1/Dβ2/Dβ3	–	–	–	–

To illustrate the extent of radioligand binding studies undertaken, the Table lists all subunit combinations containing Dα5 that were examined in transfected *Drosophila* S2 cells. Binding studies were performed with [^125^I]-α-bungarotoxin (10 nM) and [^3^ H]-epibatidine (30 nM). Combinations of *Drosophila* nAChR subunit cDNAs were transfected in the absence or presence of RIC-3 cDNA. Data indicating presence or absence of specific binding are derived from at least 3 independent experiments.

Previous studies have shown that, when co-expressed with a vertebrate β2 subunit, some *Drosophila* nAChR α-subunits can generate functional recombinant nAChRs and form high-affinity binding sites for nicotinic radioligands (see for example [16,44]). However, when Dα5 was co-expressed with vertebrate β2 in *Drosophila* S2 cells or in human tsA201 cells, no specific radioligand binding could be detected. These findings with Dα5 are similar to those conducted previously with the closely related *Drosophila* Dα6 and Dα7 subunits [15]. However, in control experiments conducted in parallel, high levels of specific radioligand binding were detected after co-expression of *Drosophila* Dα2 and Dα3 subunits with the rat β2 (Rβ2) subunit. This is in agreement with previous studies conducted with the Dα2 + Rβ2 and Dα3 + Rβ2 subunit combinations [17,45].

### Dα5/5HT3A subunit chimera

**Figure 1:**
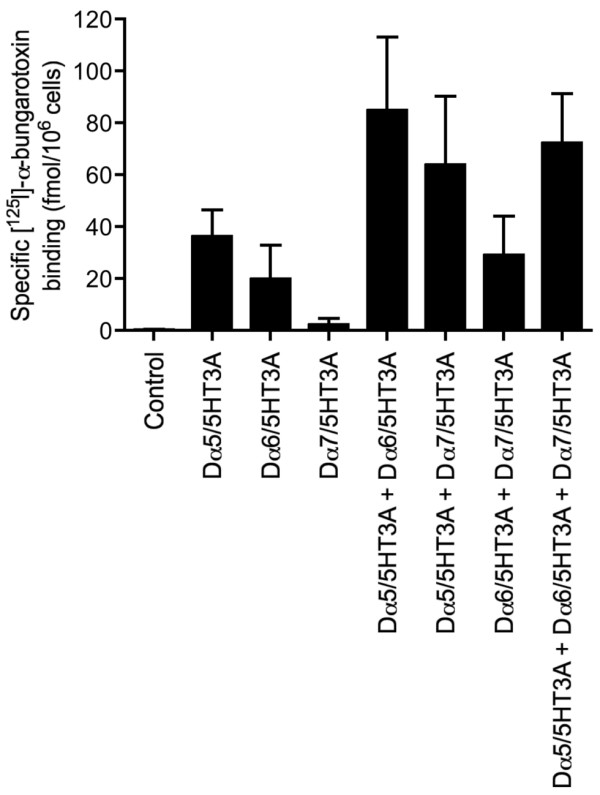
**Radioligand binding to nAChR subunit chimeras expressed in *Drosophila* S2 cells.** Cell surface [^125^I]-α-bungarotoxin binding to transiently transfected *Drosophila* S2 cells with subunit chimeras (Dα5/5HT3A, Dα6/5HT3A and Dα7/5HT3A). Experiments were performed in triplicate and are means ± SEM of 4–14 independent experiments.

As has been described previously for the Dα6 and Dα7 subunits [15], a chimera was constructed containing the N-terminal ligand-binding domain of the Dα5 subunit fused to the transmembrane and C-terminal regions of the mouse 5-HT3A subunit (5HT3A). Despite the inability of the intact Dα5 subunit to be detected by ^125^I]-α-bungarotoxin binding when expressed in *Drosophila* S2, expression of the Dα5/5HT3A chimera resulted in high levels of cell-surface ^125^I]-α-bungarotoxin binding (Figure 1). These data from recombinant subunit chimeras is consistent with evidence derived from native *Drosophila* nAChRs that Dα5 forms part of an α-bungarotoxin binding nAChR [46]. Expression studies with the intact and chimeric Dα5 subunit indicate that, in common with the Dα6 and Dα7 subunits, inefficient folding and assembly can be attributed to domains present in the C-terminal subunit domain. Similar conclusions have also been made concerning the closely related vertebrate α7 subunit [47].

The influence of co-expressing combinations of subunit chimeras was also examined. In comparisons to the level of ^125^I]-α-bungarotoxin binding detected with Dα5/5HT3A alone, higher levels of specific cell-surface binding were detected when the Dα5 chimera was co-expressed with other subunit chimeras (Dα6/5HT3A and Dα7/5HT3A; Figure 1). However, the levels of specific binding detected were not significantly higher than would have been expected from a possible additive effect of co-expressing these chimeras. Consequently, this data cannot be used as evidence to support the possibility of heteromeric co-assembly, as was the case previously for studies conducted with the Dα6 and Dα7 subunit chimeras [15].

### Heterologous expression with RIC-3

**Figure 2:**
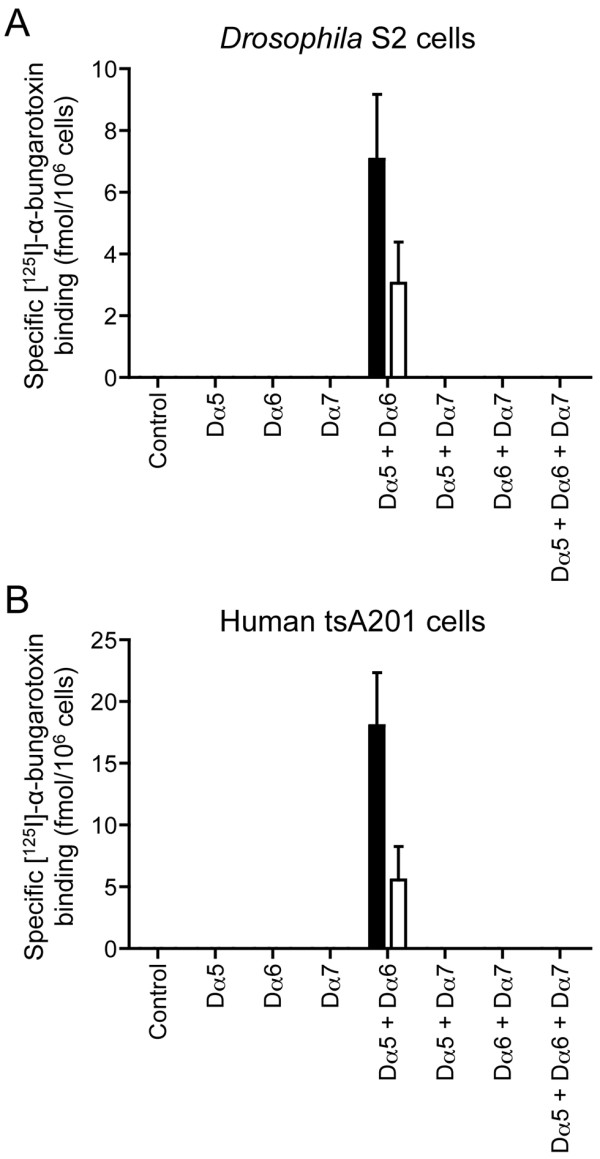
**Radioligand binding to *Drosophila* nAChR subunit combinations in cultured cell lines.** Cell surface [^125^I]-α-bungarotoxin binding to cell lines transiently transfected with combinations of Dα5, Dα6 and Dα7 subunits. In all cases, subunit combinations were co-transfected with either CeRIC-3 (filled bars) or DmRIC-3 (open bars). No specific binding was detected for any subunit combination in the absence of RIC-3 (not shown). Data are presented for *Drosophila* S2 cells (**A**) and for human tsA201 cells cultured at 25 °C (**B**). Controls represent mock-transfected cells. Experiments were performed in triplicate and are means ± SEM of 5–8 independent experiments.

Previous studies have demonstrated that the molecular chaperone protein RIC-3 can enhance maturation of several nAChRs [36]. This finding has prompted us to examine the influence of co-expressing Dα5 with RIC-3 in cultured cell lines, as we have done previously for other *Drosophila* nAChR subunits [39]. Various combinations (see Table 1 and Figure 2 for details) of Dα5, Dα6 and Dα7 were expressed with either CeRIC-3 or DmRIC-3 in both *Drosophila* S2 cells and human tsA201 cells. No specific cell-surface ^125^I]-α-bungarotoxin binding was detected when any of these subunits were expressed individually with RIC-3. However, specific binding was detected when Dα5 was co-expressed with Dα6 (Figure 2), albeit at a lower level than seen with the subunit chimeras (Figure 1). Interestingly, no specific binding was detected when these two subunits were also co-expressed with Dα7 (or other subunits; see Table 1), suggesting that Dα7 may co-assemble with Dα5 or Dα6 and, in doing so, impair receptor assembly or maturation. Similar results were obtained in both cell types examined (Figure 2), although specific binding was detected in mammalian cells only when they were cultured at a temperature lower than 37°C. As has been reported previously [15,17], lowering the temperature of transfected mammalian cells from 37°C to 25°C for 24 hours (the temperature at which *Drosophila* S2 cells are maintained) facilitates receptor assembly and cell-surface expression. As has been discussed previously with respect to insect nAChR subunits [15,17], the detection of specific radioligand binding only in mammalian cells cultured at 25°C is likely to be a consequence of more efficient subunit folding and assembly at lower temperatures.

### Expression in *Xenopus* oocytes

**Figure 3:**
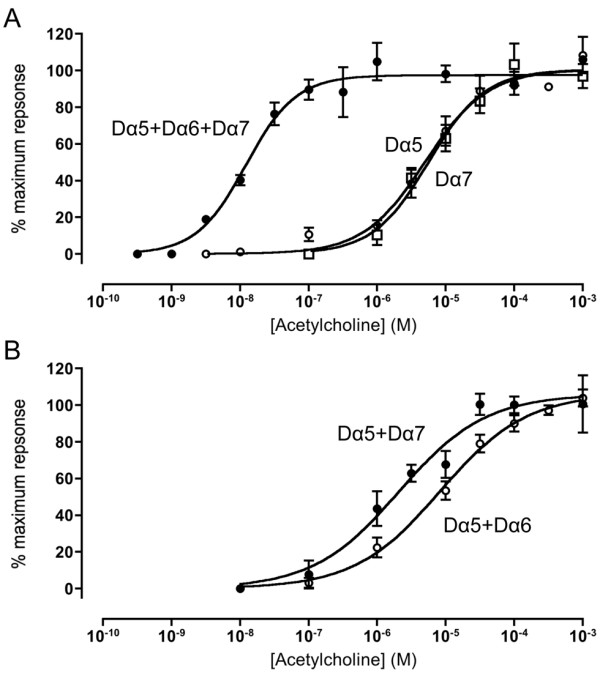
**Functional expression of *Drosophila* nAChR subunit combinations in *Xenopus* oocytes. A**) Dose–response curves for acetylcholine are shown for homomeric Dα5 nAChRs (open circles) homomeric Dα7 nAChRs (open squares) and for triplet Dα5 + Dα6 + Dα7 nAChRs (closed circles). **B**) Dose–response curves for acetylcholine are shown for heteromeric Dα5 + Dα6 nAChRs (open circles) and Dα5 + Dα7 nAChRs (closed circles) In all cases, nAChR subunits were co-expressed with CeRIC-3. Data are means ± SEM of 3–8 independent experiments.

*Xenopus* oocytes were injected with cRNA encoding various combinations of the *Drosophila* nAChR subunits Dα5, Dα6 and Dα7. No evidence of functional expression was detected for any subunit combination in the absence of co-expressed RIC-3. However, when co-expressed with CeRIC-3, functional responses to acetylcholine were detected in oocytes injected with either the Dα5 or the Dα7 subunit, indicating the presence of functional homomeric Dα5 and Dα7 nAChRs (Figure 3). However, even when co-expressed with RIC-3, functional expression was somewhat inconsistent, being observed in some but not all batches of oocytes tested (responses greater than 5 nA were observed in only about a third of the oocyte batches tested). Dose–response curves indicate that acetylcholine has a similar *EC*_50_ for these two homomeric receptor subtypes (8.8 ± 2.5 μM 6.7 ± 1.7 μM, respectively). In contrast, no functional expression was detected when Dα6 was co-expressed with CeRIC-3.

**Figure 4:**
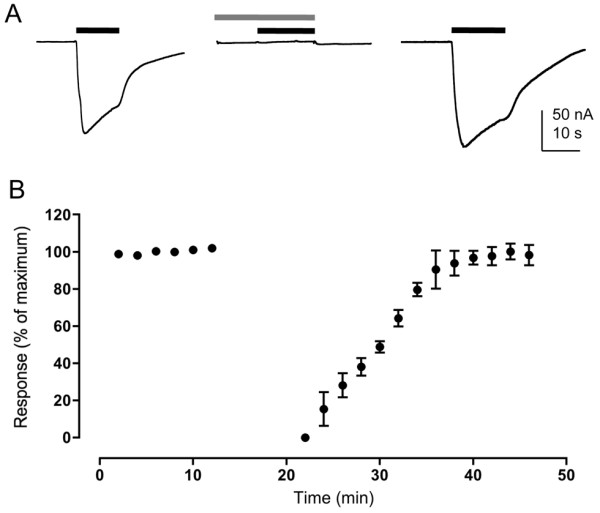
**Antagonism of Dα5 nAChRs by α-bungarotoxin. A**) Representative responses to acetylcholine (100 μM; black bar) are shown (left), together with block after a 10 min pre-incubation with α-bungarotoxin (100 nM; grey bar) (middle). Recovery from α-bungarotoxin block after 10 minutes is also illustrated (right). Data shown are for homomeric Dα5 nAChRs (co-expressed with CeRIC-3) but the results (complete block with full recovery) were observed for all subunit combinations that generated functional nAChRs (see Table 2). **B**) Data indicating the time course for recovery after a 10 min incubation with α-bungarotoxin (100 nM; illustrated by the grey bar). Data points are normalised to the maximum response prior to block by α-bungarotoxin and are means ± SEM of 3 independent experiments.

Expression of pairwise subunit combinations (with CeRIC-3) gave dose–response curves that were not significantly different (*P* > 0.05) to that of homomeric Dα5 or Dα7 nAChRs (Table 2 and Figure 3). Consequently, it was not possible to conclude whether pairwise heteromeric receptors were expressed. One pairwise combination (Dα6 + Dα7) failed to generate consistent responses, an indication that co-assembly of Dα6 with Dα7 blocks formation of functional nAChRs in oocytes. However, when all three subunits (Dα5, Dα6 and Dα7) were co-expressed with CeRIC-3, dose–response data indicated a single population of receptors with a significantly higher (ANOVA, *P* < 0.05; Student’s *t*-test *P* < 0.01) apparent affinity for acetylcholine (13.5 ± 1.7 nM; Figure 3) than that of either of the two homomeric nAChRs (Dα5 or Dα7) or any of the putative pairwise subunit combinations (Table 2). For all subunit combinations examined (see Table 2), functional responses to acetylcholine were completely blocked by a 10 min pre-incubation with 100nM α-bungarotoxin. This block was completely reversible but occurred on a slow timescale, recovery taking, typically, about 15 minutes (Figure 4). No significant differences were observed in pharmacological properties when nAChRs were co-expressed with DmRIC-3 [39], rather than CeRIC-3 (data not shown).

**Table 2:** **Functional properties of recombinant nAChRs expressed in *Xenopus* oocytes**

**Subunits**	** *EC* **_**50**_**(**μM or nM)*	**Hill slope**	** *n* ** **	** *I* **_**max**_**[ *I* **_**mean**_**]** † **(nA)**
Dα5	8.8 ± 2.5 μM	1.1 ± 0.3	6	200 [141 ± 25]
Dα6	–	–	‡	–
Dα7	6.7 ± 1.7 μM	1.0 ± 0.3	4	86 [45 ± 13]
Dα5 + Dα6	8.6 ± 2.4 μM	1.0 ± 0.1	5	47 [20 ± 8]
Dα5 + Dα7	1.6 ± 0.3 μM	1.0 ± 0.1	3	53 [39 ± 8]
Dα6 + Dα7	–	–	‡	–
Dα5 + Dα6 + Dα7	13.5 ± 1.7 nM*	1.2 ± 0.3	6	150 [107 ± 12]

Note, in all cases, nAChR subunits were co-expressed with CeRIC-3.

* Note, *EC*_50_ value for Dα5 + Dα6 + Dα7 is expressed as nM, rather than μM.

**EC_50_ and Hill slopes are means ± SEM of separate fits to dose–response curves derived from independent experiments (*n* = 3–6).

† Relatively small currents were detected with all subunit combinations, as indicated by the size of the maximum current that was detected with each subunit combination (*I*_max_) and the mean maximum current (*I*_mean_) from between 6–20 different oocytes.

‡ Subunit combinations that failed to generate functional responses are indicated by a dash. This is based on studies conducted with at least 5 batches of oocytes that generated functional nAChRs with other subunit combinations and at least 10 oocytes from each batch (*n* > 50).

## Discussion

The Dα5, Dα6 and Dα7 subunits differ from other *Drosophila* nAChR subunits in their close sequence similarity to the vertebrate α7 nAChR subunit [41,48], a subunit that is notable for its ability to form both homomeric and heteromeric nAChRs [49-52]. In addition to being one of the best characterised homomeric nAChRs, the vertebrate α7 subunit can co-assemble into heteromeric nAChRs by co-assembly with the α8 subunit (in avian speices) [50]. Although an α8 subunit is not present in mammals, recent evidence indicates that the mammalian α7 subunit can also form functional heteromeric nAChRs by co-assembly with β2 [51,52].

Relatively limited information is available about the physiological roles of the Dα5, Dα6 and Dα7 subunits in *Drosophila*, or about the role of analogous subunits in other insect species. There is, however, evidence from studies of native nAChRs in *Drosophila* that Dα5 forms part of a nAChR that is sensitive to α-bungarotoxin [46], Dα6 forms part of the spinosad-sensitive nAChR [53] and that Dα7 is required for the visually-mediated cholinergic escape response [54].

As has been discussed elsewhere [10,11], difficulties have been encountered in the efficient functional expression of insect nAChRs. Here we report the cloning of a full-length cDNA of the *Drosophila* Dα5 subunit corresponding to a previously described isoform B [41]. Other isoforms of Dα5 described previously (isoforms A and C) [41] are a consequence of alternative splicing and have fewer exons than isoform B. Isoform A lacks exon 7, which codes for part of the second transmembrane domain, whilst isoform C lacks exon 5, which codes for the region containing the extracellular Cys-loop. The cloning of the Dα5 subunit was first reported in 2002 [41] but no expression studies were described at that time. More recently, it has been reported that Dα5 does not generate functional homomeric nAChRs when expressed in *Xenopus* oocytes, even when co-expressed with RIC-3 [40]. Functional expression was, however, reported in the same study when Dα5 was co-expressed with Dα6 and RIC-3 [40]. In the present study, we have detected functional responses when Dα5 is co-expressed with Dα6 but, in contrast to the previous study [40], we have also obtained evidence for the functional expression of homomeric Dα5 nAChRs. Similarly, we have demonstrated that Dα7 can form both homomeric and heteromeric nAChRs. As far as we are aware, there have been no previous reports of the successful functional expression of Dα7, as either a homomeric or a heteromeric nAChR. Given the difficulties encountered in obtaining reproducible functional expression of insect recombinant nAChRs, it is not surprising that there may be some apparent differences in subunit combinations found to generate functional receptors in this and previous studies, particularly since the focus of the most detailed previous study was the identification of a spinosyn-sensitive nAChRs [40].

Our studies conducted in cell lines provided evidence that the pairwise combination Dα5 + Dα6 generates a high affinity radioligand binding site, a finding that agrees with previous studies demonstrating functional expression of Dα5 + Dα6 nAChRs in oocytes [40]. Interestingly, we have found no evidence of specific binding when Dα7 was co-expressed with Dα5 and Dα6 in the same cell lines. This lack of specific binding would seem to suggest that, in the two cell lines examined, co-assembly of Dα7 with the Dα5 and Dα6 subunit interferes with the formation of correctly assembled complexes. We observed a somewhat similar situation in oocytes, where expression of Dα7 alone generates functional nAChRs but it fails to do so when co-expressed with Dα6. This may reflect a tendency for Dα6 and Dα7 to assemble into non-functional complexes. The one situation where this tendency is not dominant is when all three subunits (Dα5 + Dα6 + Dα7) are co-expressed with RIC-3 in oocytes, where they are able to form a functional ‘triplet’ nAChR with high apparent affinity for acetylcholine.

The present findings suggest that the environment provided by the host cell exerts a substantial effect on the assembly of these nAChR subtypes, a phenomena that has been reported previously for other nAChRs [47,55,56]. Previous studies by another research group [40] support the conclusion that co-assembly of Dα5 + Dα6 nAChRs is somewhat inefficient. Not only was functional expression of the Dα5 + Dα6 subunit combination found to be inconsistent in the previous study, but it also appeared to be dependent on the ratio of cRNAs injected [40]. Perhaps this inconsistent functional expression reflects a tendency for some subunit combinations to assemble into non-functional complexes and that this may be more prevalent in certain subunit stoichiometries. It is possible that, in the native cellular environment, factors determining efficiency of subunit assembly and maturation may differ, perhaps as a consequence of a different array of endogenous chaperone proteins. This conclusion is supported by previous studies that have indicated that influence of RIC-3 on maturation of nAChRs is influenced by the host cell [39] and may help to explain the differences that we have observed in the ability of some subunit combinations to assemble into nAChRs in different expression systems.

The data obtained from expression studies in *Drosophila* and human cell lines is broadly similar. However, successful expression in human cells required incubation at a temperature lower than they would normally be maintained at (25°C, rather than 37°C) [note: *Drosophila* S2 cells are routinely maintained at 25°C]. Previous studies have demonstrated that the folding and assembly of the nAChRs from insects [17] and from some other non-insect species, such as the cold-water ray *Torpedo*[57], can be influenced by temperature. This temperature dependence appears to be a consequence of inefficient protein folding and/or subunit assembly at higher temperatures. Previously, due to difficulties in expression of Dα6 and Dα7 nAChR subunits, we examined the ability of subunit chimeras to assemble into complexes capable of binding ^125^I]-α-bungarotoxin [15]. From such studies, it was possible to conclude that the Dα6 and Dα7 subunits were capable of heterometic co-assembly. In the present study the data from subunit chimeras is less clear cut. Although higher levels of ^125^I]-α-bungarotoxin were seen consistently when the Dα5 chimera was co-expressed with either the Dα6 and Dα7 chimeras, it was not clear in all cases whether this was greater than an additive effect. Nevertheless these findings are consistent with the conclusion that Dα5 is able to co-assemble into heteromeric complexes. For all subunit combinations examined, responses to acetylcholine were completely blocked by α-bungarotoxin, a finding that is consistent with previous studies conducted with native nAChRs purified from *Drosophila* which demonstrated that Dα5 is part of an α-bungarotoxin binding nAChR [46].

As mentioned above, a previous study has reported the functional expression of heteromeric Dα5 + Dα6 nAChRs (co-expressed with RIC-3) in *Xenopus* oocytes and also the inability of either Dα5 or Dα6 to form functional homomeric nAChRs [40]. Significantly, the authors of this earlier study describe substantial difficulties in achieving reliable functional expression. In the present study, despite demonstrating the functional expression of several combinations of the Dα5, Dα6 and Dα7 subunits, we have also encountered a much lower success rate than is typically achieved with other nAChRs. In both transfected cell lines and in *Xenopus* oocytes, we occasionally failed to detect evidence of radioligand binding or functional expression, despite success with other nAChRs that were expressed as positive controls (for example the mammalian α7 nAChR). The difficulties that we and others have encountered may be associated with a tendency for these subunits to co-assemble into non-functional complexes. It is possible that this may reflect a requirement for additional chaperone proteins. Indeed, a study conducted with a *C. elegans* nAChR has demonstrated a requirement for three different chaperone proteins for efficient functional heterologous expression [58].

## Conclusions

In summary, whereas it has been reported previously that Dα5 and Dα6 can form a functional heteromeric nAChR (albeit inefficiently) when expressed in *Xenopus* oocytes [40], this is the first evidence that either Dα5 or Dα7 can form functional homomeric nAChRs. It is also the first demonstration that Dα7 can form a functional heteromeric nAChR. Of particular interest is the evidence that the three subunits examined in this study can co-assemble to form a functional triplet (Dα5 + Dα6 + Dα7) nAChR with a high apparent affinity for acetylcholine.

## Methods

### Plasmids and cRNA synthesis

Subcloning of *Drosophila* nAChR subunit cDNAs Dα1(ALS), Dα2(SAD), Dα3, Dα4, Dα6, Dα7, Dβ1(ARD), Dβ2(SBD) and Dβ3 [alternative subunit nomenclature in parenthesis] into expression vectors pRmHa3 and pRK5 has been described previously [15,17,59,60]. Construction and subcloning of Dα6/5HT3A and Dα7/5HT3A subunit chimeras has also been described previously [15]. For expression studies in *Xenopus* oocytes, subunit cDNAs were subcloned into pGEMHE [61] downstream of the SP6 promoter. Plasmid constructs (pGEMHE) containing nAChR subunit cDNAs were linearized with *Nhe*I and purified with QIAQuik PCR purification kit (Qiagen). *In vitro* synthesis of cRNA was performed using mMessage mMachine SP6 transcription kit (Ambion). *C. elegans* RIC-3 (CeRIC-3) cDNA [35] was provided by Millet Treinin (Hebrew University, Israel). The *D. melanogaster* RIC-3 (DmRIC-3) cDNA used in this study corresponds to the previously described splice variant DmRIC-3^7a,9^[39].

### Molecular cloning of Dα5

Oligonucleotide primers were synthesized which correspond to the predicted 5′ and 3′ untranslated regions of transcript CG32975 identified by the GadFly *Drosophila* genome annotation project (flybase.org). A first-strand cDNA synthesis kit (G.E. Healthcare) was used to isolate cDNA from *Drosophila melanogaster* embryo Poly A + RNA (Clontech). A 2425 bp fragment was amplified using KOD hot start polymerase (Novagen) and was subcloned into plasmid pCRII (Invitrogen). The cDNA construct was sequenced and found to correspond to the previously identified isoform B [41] [note: isoforms A and C show alternative splicing and have fewer exons than isoform B]. The cDNA fragment was subcloned into the *Eco*RI site of pRmHa3 and pGEMHE to create pRmHa3-Dα5 and pGEMHE-Dα5.

### Construction of Dα5/5HT3A chimera

To construct a Dα5/5HT3A chimera, a similar approach was used to that described previously for the *Drosophila* Dα6 and Dα7 subunits [15] and for mammalian nAChR subunits [47,62]. A *Bcl*I site was introduced into pRmHa3-Dα5 by means of the QuikChange site-directed mutagenesis method (Stratagene) at a position equivalent to V201 in the previously described mammalian α7/5HT3A chimera [62]. The C-terminal region of Dα5 was removed by digestion with *Bcl*I and *Sma*I and the corresponding region of the mouse 5HT_3A_ subunit [63] ligated to create the construct pRmHa3-Dα5/5HT3A. The chimeric cDNA was subcloned into plasmid pRK5 by excising the construct from pRmHa3 with restriction enzymes *Eco*RI and *Xba*I to create pRK5-Dα5/5HT3A.

### Heterologous expression in cultured cell lines

Schneider’s *Drosophila* S2 cells [64] were obtained from Dr Thomas Bunch, University of Arizona, and grown in Shields and Sang M3 medium (Sigma) containing 12.5% heat inactivated foetal calf serum (First Link), 100U/ml penicillin and 100 μg/ml streptomycin (Invitrogen) at 25°C. Exponentially growing S2 cells were transfected by a modified calcium phosphate method as described previously [65]. Cells were transiently transfected with plasmid pRmHa3 were induced by the addition of CuSO_4_ (0.6 mM) for 24 h. Human kidney tsA201 cells [66] were obtained from Dr William Green, University of Chicago, and cultured in Dulbecco’s modified Eagle’s medium (Invitrogen) containing 10% foetal calf serum (First Link) and 100U/ml penicillin and 100 μg/ml streptomycin (Invitrogen). Cells were maintained in a humidified incubator containing 5% CO_2_ at 37°C. Cells were transfected using Effectene (Qiagen) according to the manufacturer’s instructions and incubated overnight at 37°C. To facilitate efficient folding and assembly of *Drosophila* nAChR subunits, cells were incubated at 25°C for a further 24 hours, before being assayed for radioligand binding.

### Radioligand binding

^3^ H]-epibatidine (56.31 Ci/mmol) and ^125^I]-α-bungarotoxin (2200 Ci/mmol) were purchased from Perkin Elmer. ^3^ H]-methyllycaconitine (100 Ci/mmol) was purchased from American Radiolabeled Chemicals. Radioligand binding to transiently transfected S2 or tsA201 cells (both whole cell or membrane preparations) using tritiated ligands has been described previously [45]. Samples were assayed by filtration onto Whatman GF/B filters pre-soaked in 0.5% polyethylenimine (PEI) followed by rapid washing using a Brandel cell harvester. Samples assayed using the ligand ^125^I]-α-bungarotoxin were incubated in buffer containing 0.5% BSA and harvested onto Whatman GF/A filters pre-soaked in 0.5% PEI, as described previously [15]. Preliminary experiments were carried out to ensure that incubation times were long enough to enable radioligand binding to reach equilibrium. Amounts of total cellular protein were determined by a Bio-Rad DC protein assay using bovine serum albumin standards.

### Oocyte electrophysiology

Adult female *Xenopus laevis* frogs were obtained from the European *Xenopus* Resource Centre (University of Portsmouth). Oocytes were isolated and defolliculated as described previously [67] following procedures that have been approved by both UCL’s Biological Services Management Group and the UK Home Office (under licences PIL70/23585 and PPL70/06819). For heterologous expression, cRNA (6–12 ng) was injected into the oocyte cytoplasm in a volume of 32.2 nl, using a Nanoject II microinjector (Drummond Scientific). Experiments were performed, typically, 2–4 days after injection of oocytes. Two electrode voltage-clamp recordings (with the oocyte membrane potential held at -60 mV) were performed essentially as described previously [67], using a Warner Instruments OC-725 C amplifier (Harvard Apparatus), PowerLab 8SP and Chart 5 software (AD Instruments). Agonists were applied to oocytes using a BPS-8 solenoid valve solution exchange system (ALA Scientific), controlled by Chart software. Data were analyzed using GraphPad Prism software. For multiple comparisons, statistical significance was determined by ANOVA with Tukey’s post-hoc test. Additional pair-wise comparisons were performed by Student’s *t*-test.

## Abbreviations

nAChR, nicotinic acetylcholine receptor.

## Competing interests

The authors declare that they have no competing interests.

## Authors’ contributions

SJL performed the experimental work (molecular biological, pharmacological and electrophysiological), interpreted the data and helped to write the manuscript. TC performed some of the molecular biological work. JG was involved in planning experiments and assisted in interpretation of the data. NSM designed the study, assisted in interpreting the data and helped to write the manuscript. All authors read and approved the final manuscript.
